# γ-Aminobutyric acid treatment induced chilling tolerance in postharvest peach fruit by upregulating ascorbic acid and glutathione contents at the molecular level

**DOI:** 10.3389/fpls.2022.1059979

**Published:** 2022-12-07

**Authors:** Chujiang Zhou, Wanqi Dong, Shuwan Jin, Qingli Liu, Liyu Shi, Shifeng Cao, Saisai Li, Wei Chen, Zhenfeng Yang

**Affiliations:** ^1^ College of Food Science and Technology, Shanghai Ocean University, Shanghai, China; ^2^ College of Biological and Environmental Sciences, Zhejiang Wanli University, Ningbo, China

**Keywords:** γ-aminobutyric acid, ascorbic acid, glutathione, chilling injury, peach

## Abstract

Peach fruit was treated with 5 mM γ-aminobutyric acid (GABA) to further investigate the mechanism by which GABA induced chilling tolerance. Here, we found that GABA not only inhibited the occurrence of chilling injury in peach fruit during cold storage but also maintained fruit quality. Most of the ascorbic acid (AsA) and glutathione (GSH) biosynthetic genes were up-regulated by GABA treatment, and their levels were increased accordingly, thus reducing chilling damage in treated peaches. Meanwhile, the increased transcript of genes in the AsA-GSH cycle by GABA treatment was also related to the induced tolerance against chilling. GABA treatment also increased the expression levels of several candidate ERF transcription factors involved in AsA and GSH biosynthesis. In conclusion, our study found that GABA reduced chilling injury in peach fruit during cold storage due to the higher AsA and GSH contents by positively regulating their modifying genes and candidate transcription factors.

## Introduction

Peach (*Prunus persica* (L.) Batsch) is a typical climacteric fruit. Low-temperature storage can inhibit its respiratory and endogenous ethylene peak during postharvest storage, which slows the process of postharvest ripening and senescence (Mahmud et al., 2017). However, when exposed to unsuitable cold temperatures, peach fruit is highly susceptible to chilling damage. Chilling symptoms such as fruit flocculation, flesh browning, and juice reduction can occur under low-temperature storage, resulting in loss of fruit flavor and texture, seriously affecting its edible quality and market economic value.

γ-Aminobutyric acid (GABA) is a naturally occurring amino acid in plants and animals (Nicolas and Hillel, 2004). In plants, GABA plays a role under stress conditions by regulating the activities of related enzymes or the synthesis of metabolites (Bao et al., 2015; Ramesh et al., 2017; Li et al., 2021). For instance, exogenous GABA inhibited chilling injury in tomatoes by scavenging reactive oxygen species (ROS) and increasing antioxidant enzyme activity (Malekzadeh, 2015). Wang et al. (2014a) found that exogenous GABA treatment induced resistance against chilling in melon by promoting polyamine synthesis Additionally, exogenous GABA treatment induced the synthesis of endogenous GABA in bananas (Wang et al., 2014c), persimmon (Niazi et al., 2021) and cucumber (Malekzadeh et al., 2017), thereby increasing the cold resistance and prolonging their low-temperature storage time. There are also many studies on GABA in peach fruit. For example, Aghdam et al. (2016) found that GABA can effectively maintain the content of ascorbic acid, total phenol, flavonoids, and other substances in peach fruit and the ability to eliminate free radicals. Recently, a study found that GABA can improve the cold resistance of peach fruits by promoting the methionine sulfoxide reductase thioredoxin reductase system (Jiao, 2022).

When plants are subjected to stress, ROS are produced rapidly, resulting in tissue damage (Baxte et al., 2014). A complete antioxidant system in plants can rapidly reduce the level of ROS to the average level. AsA and GSH are important antioxidants that can scavenge excess ROS and reduce damage caused by different stresses (Alscher et al., 1997; Song et al., 2016; Hasanuzzaman et al., 2019; Ma et al., 2019). Therefore, investigating the formation and regulation of AsA and GSH is of great significance in improving fruit quality and enhancing stress resistance in postharvest fruit.

Significant progress has been made in investigating the AsA biosynthetic pathway in plants. Four possible pathways of AsA biosynthesis in plants have been proposed. Among these pathways, the L-galactose pathway plays a leading role in plant AsA synthesis (Wheeler et al., 1998), while other pathways such as D-galacturonate, L- gluose and Myo-inositol supplement it (Agius et al., 2003; Lorence et al., 2004; Wolucka et al., 2005). All the genes encoding the enzymes involved in this pathway have been identified, including phosphomannomutase (PMM), GDP-D-mannose pyrophorylase (GMP), GDP-D-mannose-3’,5’-heteroisomerase (GME), GDP-L-galactose phophorylase (GGP), L-galactose-1-phosphate phophoratase (GPP) and L-galactose dehydrogenase (GalDH). PMM and GMP catalyze the synthesis of GDP-D-mannose, providing a substrate for AsA synthesis. GME catalyzes the conversion of GDP-D-mannose to GDP-L- galactose, which is the first step of AsA biosynthesis at the glycoside level. GGP catalyzes GDP-L-galactose to produce GDP-L- galactose -1- phosphate (Wheeler et al., 1998), which is one of the vital catalytic enzymes in the L-galactose pathway. GalDH catalyzes L- galactose to produce L- galactose -1,4-lactone, one of the rate-limiting enzymes in the L-galactose pathway. The specific catalytic steps of the other three AsA synthetic pathways in plants are still at the stage of speculation because the enzyme genes in these pathways have not been fully identified except for inositol oxygenase (MXIO) and D-galactonic acid dehydrogenase (GalUR) (Wolucka and Van Montagu, 2003; Lorence et al., 2004).

GSH biosynthesis is mainly catalyzed by γ-glutamylcysteine ligase (GCS) and glutathione synthase (GS). First, L-glutamic acid and cysteine are catalyzed by GCS to synthesize γ-glutathione cysteine. Then, under the catalysis of glutathione synthase (GS), glycine is added to the C-terminus of γ-glutathione cysteine to produce glutathione.

Our previous study showed that GABA could inhibit chilling injury of peach fruit during cold storage (Shang et al., 2011; Yang et al., 2011). However, the transcriptional activity of genes involved in the metabolism of AsA and GSH remained unstudied. Therefore, this study further investigated the mechanism by which GABA induced cold tolerance in chilled peach fruit by influencing AsA and GSH levels at the molecular level. In addition, we also analyzed the expression of three ERF transcription factors that may be related to ASA-GSH metabolism.

## Materials and methods

### GABA treatment

Peach fruit (*Prunus. Persica* Batsch cv. Hujing) were harvested at commercial maturity (120 days after flowering) from experimental farm of Fenghua Peach Fruit Research Institute (Ningbo, China). The peaches were picked and transported to laboratory quickly and then selected in uniform size and randomly divided into two groups with sixty-six fruit in each group. The conforming and undamaged fruit were selected and randomly divided into two groups According to our previous study (Yang et al., 2011), the fruit in first group were immersed in a solution of 5 mM GABA for 20 min, while the other was immersed in water for 20 min as control. Afterwards, both groups of fruit was air-dried for approximately two hours and transfer to 4 °C stored for thirty-five days. Eight peaches from each sampling site in each group were frozen in liquid nitrogen and stored in an ultra-low temperature refrigerator after fruit firmness, total soluble solid (TSS) and relative electrical conductivity were determined. From three weeks of cold storage, another six peaches were taken at each sampling point and transferred from 4 °C to 20 °C for three days to simulate the shelf environment and evaluated the chilling injury index. The whole experiment was repeated three times.

### Determination of chilling injury index, fruit firmness, total soluble solid and relative electrical conductivity

The degree of chilling was visually assessed on the mesocarp surface, following a double cut parallel to the axial diameter. The extent of flesh browning was divided into four classes: 0, no browning; 1, browning covering <25% of the cut surface; 2, browning covering ≥25% but <50% of cut surface; 3, browning covering ≥50%. CI index was calculated using the following formula: Chilling injury index = Σ [(browning level) × (number of fruit at the browning level)]/(total number of fruit in the treatment).

A texture analyzer (TMS-Touch, US) with a 7.5 mm diameter probe at a rate of 10 mm s^−1^ was used to measure the firmness of both sides of peeled peaches. TSS was measured using a handheld refractometer (G-won, GMK-701AC, Korea).

Electrolyte leakage was evaluated according to Zhao et al. (2019). Cylinders of peach tissue were excised with a 5 mm diameter stainless steel cork borer from the equatorial region. After rinsing three times (2 min to 3 min) with deionized water, 12 small discs (0.1 cm thick, 0.5. cm diameter) were incubated in 25 mL of deionized water at 20°C, followed by shaking for 30 min. Electrolyte leakage of the solution was measured using a conductivity meter (DDS-11A; Shanghai, China). The solution was then heated to 100°C for 15 min and quickly cooled. The total electrolytes of the solution were then measured again. Relative leakage was expressed as a percentage of the total electrolyte leakage.

### Determination of levels of AsA, GSH and GSSG

A method of Hu et al. (2016) was used to measure AsA content. Approximately 0.1 g of frozen sample were ground in liquid nitrogen. The homogenates were then extracted with 5 mL of 5% (w/v) trichloroacetic acid (TCA) and centrifuged at 12,000 g at 4° for 15 min to obtain supernatant for AsA determination. Took 1 ml of supernatant after centrifugation at 4°C and added 0.5 ml of 0.4% phosphoric acid ethanol solution, 1.0 ml of 0.5% phenanthroline ethanol solution, 0.5 ml of 0.03% FeCl_3_ ethanol solution. After mixing, put the solution at 30°C and reacted for 60 min, then measured the absorbance at 534 nm. Two grams of peach fruit was grounded in liquid nitrogen with 2 mL of extraction solution and then centrifuged at 12000 g for 10 min at 4°C. The supernatant was collected for GSH and GSSG determination with a glutathione kit (Beijing Solabao Technology Co., Ltd., Ningbo, China).

### Gene expression analysis

Extraction and reverse transcription of total RNA were performed according to the method of Zhang et al. (2021). Gene expression analysis was performed on a StepOnePlus™ real-time PCR instrument (BIO-RAD, Hercules, California, USA) using SYBR Green I Master Mix (Vazyme, Nanjing, Jiangsu, China) and specific primers (**Supplementary Table 1**). The real-time fluorescence quantitative PCR reaction system is 6.25 μL GreenMaster Mix, 0.5 μL upstream primer, 0.5 μL downstream primer, 5.25 μL template DNA. Reaction range Sequence: 95 °C pre denaturation for 5 min; deformation at 95 °C for 10 s, annealing at 60 °C for the 30 s, and cycling for 40 times. Gene expression was calculated using the 2^-ΔCt^ method.

### Statistical analysis

Experimental data were analyzed using GraphPad Prism 9 software. Differences between control and treatment were tested using multiple t tests (* P < 0.05, ** P < 0.01, and *** P < 0.001).

## Results

### Effect of GABA treatment on CI index in peaches

**Figure 1:**
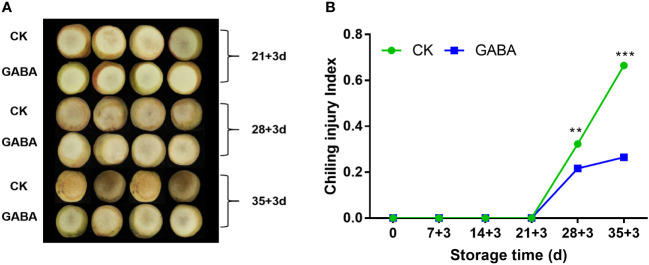
Effect of GABA treatment on the appearance **(A)** and chilling injury index **(B)** of peach stored at 4°C. Asterisks indicate significant differences between CK and GABA treatment (*p < 0.05, **p < 0.01, and ***p < 0.001).

CI of the non-treated peach fruit increased sharply after three weeks of cold storage. GABA treatment reduced the occurrence of Cl, and the index was considerably lower than that of untreated peach after 28 d of storage (**Figures 1A, B**). In the 35 d, CI of peach treated with GABA was 44.2% lower than the untreated group.

### Effect of GABA treatment on fruit firmness, TSS and relative electrical conductivity in peaches

**Figure 2:**
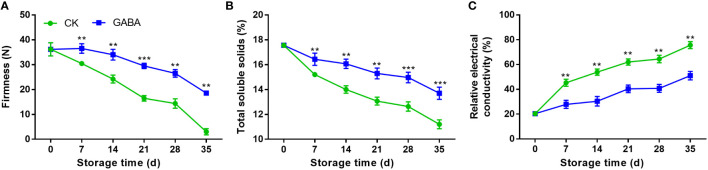
Effect of GABA treatment on fruit firmness **(A)**, total soluble solids **(B)** and relative electrical conductivity **(C)** of peach stored at 4°C. Asterisks indicate significant differences between CK and GABA treatment (*p < 0.05, **p < 0.01, and ***p < 0.001).

Both GABA-treated and non-treated peaches exhibited a decline in fruit firmness with storage time. Compared to the control group, fruit treated with GABA maintained a greater level of firmness over the whole storage (**Figure 2A**). TSS contents of peach fruit gradually decreased along with storage time, GABA-treated fruit experienced higher TSS levels than the controls (**Figure 2B**). Relative electrical conductivity increased rapidly in control peaches during the cold storage, which was inhibited with GABA treatment (**Figure 2C**).

### Effect of GABA treatment on levels of AsA, GSH and GSSG in peaches

**Figure 3:**
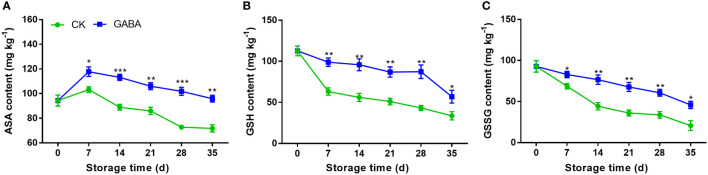
Effect of GABA treatment on AsA **(A)**, GSH **(B)** and GSSG **(C)** contents of peach stored at 4°. Asterisks indicate significant differences between CK and GABA treatment (*p < 0.05, **p < 0.01, and ***p < 0.001).

At the beginning of cold storage, AsA levels increased, but then decreased until the end (**Figure 3A**). On day 7, the contents of AsA content in both control and GABA treated peaches reached the maximum values. After that, the content in the untreated group decreased rapidly, while the GABA-treated group maintained a higher level. Overall, the AsA content in the GABA-treated peach were higher than those of the controls throughout the whole storage. The levels of GSH and GSSG in the non-treated peach decreased gradually within the storage. The results showed that GABA treatment up-regulated peach fruit’s GSH and GSSG contents throughout storage (**Figures 3B, C**).

### Effect of GABA treatment on expression of genes involved in AsA biosynthesis in peaches

**Figure 4:**
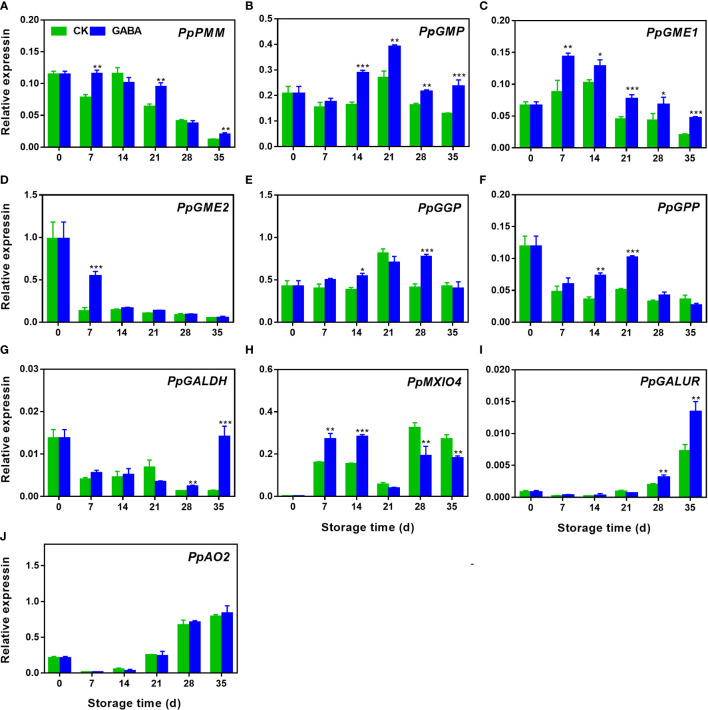
Effect of GABA treatment on expression of genes involved in AsA biosynthesis of peach stored at 4°C. Expression levels of *PpPMM***(A)**,*PpGMP***(B)**, PpGME1 **(C)**, PpGME2 **(D)**, PpGGP **(E)**, PpGPP **(F)**, PpGALDR **(G)**, PpGALUR **(H)**, PpMXIO4 **(I)** and PpAO2 **(J)** were analysed. Asterisks indicate significant differences between CK and GABA treatment (*p < 0.05, **p < 0.01, and ***p < 0.001).

During storage, the expression of *PpPMM* gradually decreased, GABA treatment induced its expression on day 7, 21 and 35 (**Figure 4A**). The transcripts of *PpGMP* and *PpGME1* increased firstly and then declined thereafter. GABA upregulated *PpGMP* after fourteen days of storage but *PpGME1* during the whole storage (**Figures 4B, C**). The expression of *PpGME2* declined dramatically during the first seven days of storage and then remained unchanged. A higher level of its expression was observed on day 7 in GABA-treated peaches (**Figure 4D**). The transcript abundance of *PpGGP* and *PpGPP* increased during first 21 d of storage followed by a decline during the remaining storage time. Higher transcripts of *PpGGP* were observed on day 14 and 28 of storage after GABA treatment (**Figure 4E**). The treatment also upregulated *PpGPP* expression on day 14 and 21 as compared to the controls (**Figure 4F**). A decrease of *PpGALDH* expression was observed in non-treated peaches, GABA treatment induced it after 28 d of storage (**Figure 4G**). GABA treatment also upregulated the expression of *PpMXIO4* during the first 14 d of storage but inhibited it after 28 d (**Figure 4H**). Regarding to the gene *PpGALUR* in D-galacturonate pathway and gene *PpAO2* in AsA degradation, both of their expression increased within the storage in peaches, GABA induced *PpGALUR* after 28 d of storage, however, no difference was observed in *PpAO2* expression between the control and treated peaches (**Figures 4I, J**).

### Effect of GABA treatment on expression of genes involved in GSH biosynthesis in peaches

**Figure 5:**
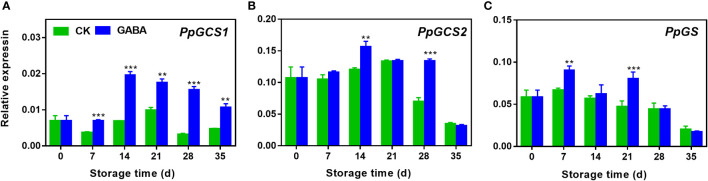
Effect of GABA treatment on expression of genes involved in GSH biosynthesis of peach stored at 4°C. Expression levels of *PpGCS1* **(A)**> *, PpGCS2* **(B)** and *PpGS* **(C)** were analysed. Asterisks indicate significant differences between CK and GABA treatment (*p < 0.05, **p < 0.01, and ***p < 0.001).

As shown in **Figure 5**, the transcripts of all three genes such as *PpGCS1*, *PpGCS2* and *PpGS* increased firstly and declined gradually thereafter. GABA treatment induced *PpGCS1* expression in peach during the whole storage (**Figure 5A**). Higher transcripts of *PpGCS2* were observed on day 14 and 28 of storage after GABA treatment (**Figure 5B**). The treatment also upregulated *PpGS* expression on day 7 and 21 as compared with the control (**Figure 5C**).

### Effect of GABA treatment on expression of genes involved in AsA-GSH recycling in peaches

**Figure 6:**
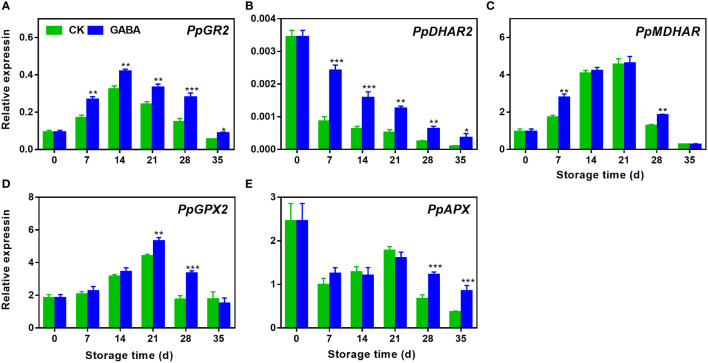
Effect of GABA treatment on expression of genes involved in ASA-GSH cycle of peach stored at 4°C. Expression levels of PpGR2 **(A)**, PpDHAR2 **(B)**, PpMDHAR **(C)**, PpGPX2 **(D)** and PpAPX **(E)** were analysed. Asterisks indicate significant differences between CK and GABA treatment (*p < 0.05, **p < 0.01, and ***p < 0.001).

Expression of *PpGR2* increased initially and then began to decrease while the expression of *PpDHAR2* decreased gradually during storage. GABA promoted the transcription level of both genes over the storage (**Figure 6A, B**). During the first 21 d of storage, *PpMDHAR* and *PpGPX2* transcript abundance increased but then decreased quickly. Up-regulations of *PpMDHAR* was observed on day 7 and 28 in GABA-treated peaches (**Figure 6C**) and GABA also induced *PpGPX2* expression on day 21 and 28 (**Figure 6D**). Compared with the control peach, there was no difference in the levels of *PpAPX* expression between the treated and control fruit in the early storage stage. However, GABA treatment increased the abundance of *PpAPX* transcripts after 28 d of cold storage (**Figure 6E**).

### Effect of GABA treatment on expression of ERF transcription factors in peaches

**Figure 7:**
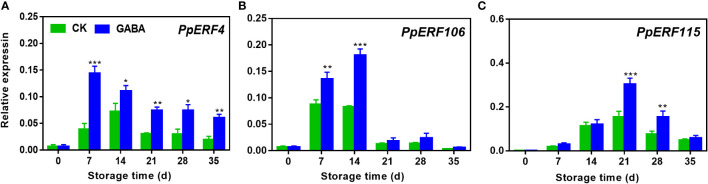
Effect of GABA treatment on expression of *ERF* transcription factors of peach stored at 4°C. Expression levels of PpERF4 **(A)**, PpERF106 **(B)** and PpERF115 **(C)** were analysed. Asterisks indicate significant differences between CK and GABA treatment (*p < 0.05, **p < 0.01, and ***p < 0.001).

The transcript levels of all the three candidate ERF transcription factors exhibited a sharp increase and subsequent decrease during the cold storage in both treated and non-treated peach. GABA treatment increased *PpERF4* expression during the whole storage (**Figure 7A**). Higher transcripts of *PpERF106* were observed on day 7 and 14 of storage after GABA treatment (**Figure 7B**). The treatment also upregulated *PpERF115* expression on day 21 and 28 as compared to the controls (**Figure 7C**).

## Discussion

GABA is a non-protein amino acid that accumulates rapidly in plants when stressed (Ramos-Ruiz et al., 2019). It plays a crucial role in regulating pH in the cytosol, controlling carbon and nitrogen metabolism, and protecting cells from biotic and abiotic stresses (Hijaz et al., 2018). In recent years, there have also been many related researches on GABA-inducing chilling tolerance in post-harvest in fruit and vegetables (Ali et al., 2022; Fan et al., 2022; Jiao, 2022). Same as our previous studies (Yang et al., 2011), here we also found GABA could effectively reduce chilling incidence and maintain fruit quality during postharvest storage.

AsA plays a critical role in oxidative stress defense and membrane degradation in plants. Increased plant resistance against cold stress due to enhanced hydrogen peroxide scavenging system by AsA, is widely reported in postharvest bananas, kiwifruit and peach (Wang et al., 2014b; Yang et al., 2016; Lo’Ay and El-Khateeb, 2018). Here, our research found the AsA levels in GABA-treated peach increased during storage which was crucial to relieve oxidative stress and reduce the chilling injury in peach fruit.

The biosynthesis of AsA in plant is mainly divided into four pathways, namely L-galactose (Wheeler et al., 1998), D-galacturonate (Agius et al., 2003), L-glucose (Wolucka et al., 2005) and myo-inositol (Lorence et al., 2004). Among them, the L-galactose pathway has been officially recognized as the leading synthetic pathway of AsA biosynthesis (Zhang et al., 2015). Gómez-García and Ochoa-Alejo (2016) found that it was the primary mechanism for AsA biosynthesis in fruit and leaves of peppers. Li et al. (2011) revealed that the expression of four L-galactose genes such as *MdGME*, *MdGPP* and *MdGaLDH*) in young apple fruit was correlated well with the AsA content. In our current study, most genes of the L-galactose pathway were up-regulated by GABA treatment, consequently responsible for the corresponding increase in AsA levels to some extent.

Besides the L-galactose pathway, the D-galacturonate pathway is also reported to be the major pathway for AsA biosynthesis in plants, in which *GALUR* is a crucial enzyme (Agius et al., 2003), converting D-galacturonate into L-galactose acid (Badejo et al., 2012). In Arabidopsis overexpressed with strawberry *FaGALUR* gene ectopically, AsA content was increased (Wolucka and Van Montagu, 2003). However, there was no apparent relationship between AsA content and *AdGALUR* expression in kiwifruit (Bulley et al., 2009). Our experiment here showed that GABA treatment did not enhance the transcription level of *PpGALUR* in the early stage of storage but considerably increased expression was observed at the end of storage. Therefore, we speculated that *PpGALUR* in GABA-treated peach might play a role in inducing AsA accumulation during the late period of storage.

In addition to the two AsA biosynthetic pathways described above, we also studied the transcriptional abundance of some genes in the myo-inositol and degradation pathways. As a key enzyme in the myo-inositol pathway, the role of *MIOX* in the plant AsA biosynthesis pathway is still unclear (Munir et al., 2020). Previous studies have suggested that overexpression of *AtMIOX* in Arabidopsis increased the AsA level by 2-to-3 fold (Lorence et al., 2004). A study has shown, however, that *AtMIOX* regulated the Myo-inositol level without increasing AsA content (Endres and Tenhaken, 2009). In our present study, the different responses of *PpMIOX4* transcripts in response to GABA treatment were observed indicating the precise mechanism of *PpMIOX4* in AsA biosynthesis in peach fruit needs to be further explored. In respect to the AsA degradation, no differential expression of *PpAO2* was found in peaches treated with GABA. Therefore, we believed that the GABA signal was not involved in the regulation of AsA degradation.

GSH is another well-known antioxidant in plants that can reduce the accumulation of ROS produced in various adversity stresses (Chen and Yang, 2020). It was found that increasing GSH content could reduce chilling injury in postharvest fruit such as iwifruit, pears and bell pepper (Wei et al., 2019; Yao et al., 2021; Jiao et al., 2022). Similarly, in GABA-treated peach treatment, higher GSH content was observed, which was also related to the induced chilling tolerance in peach treated with GABA.

It is well documented that GSH biosynthesis is mainly a free amino acid-dependent enzymatic synthesis of ATP and Mg^2+^. γ-Glutamylcysteine ligase (GCS) is a key rate-limiting enzyme in GSH biosynthesis catalyzing γ-COOH and cysteine to glutamate -NH-binding acid while consuming ATP to generate glutamylcysteine (γ-EC) (Noctor et al., 2012). Then, glutathione synthase (GS) catalyzes γ-EC to generate GSH (Jobbagy et al., 2019). In our experiments, GABA treatment increased the expression of all of the three GSH biosynthetic genes in peach, thus inducing GSH accumulation.

The AsA-GSH cycle is an essential H_2_O_2_ scavenging system in plants (Hasanuzzaman et al., 2019). AsA and GSH are important non-enzymatic antioxidants in this cycle, and their redox states can represent the redox states in the cellular environment (Wang et al., 2013). GPX, MDHAR, DHAR, GR and APX are critical enzymes in the metabolism of the AsA-GSH cycle. GPX has a defensive role against oxidative damage in plant cells (Lushchak, 2012). MDHAR and DHAR can reduce MDHA and DHA respectively to form AsA, ensuring the efficient regeneration of AsA, while GSH is oxidized to GSSG (Aravind and Prasad, 2005; Ma et al., 2019). GR can use NAD (P) H as an electron donor to regenerate GSSG into GSH and maintain the reduced state of GSH. APX can use AsA as an electron donor to reduce H_2_O_2_ to H_2_O, and remove excess H_2_O_2_ in cells (Yin et al., 2010). Studies have shown that Glycine betaine treatment reduced oxidative stress damage and improved cold resistance by regulating the metabolism of the AsA-GSH cycle in plants (Einset and Connolly, 2009). In peach, we found that GABA treatment up-regulated the expression of *PpGR2, PpDHAR2*, *PpMDHAR*, *PpGPX2* and *PpAPX* genes in the AsA-GSH cycle during cold storage, which was similar to the results obtained by Wang et al. (Wang et al., 2016), who reported that Glycine betaine treatment also induced associated genes in the AsA-GSH cycle, thus improving cold resistance in pepper fruit. Our results showed that GABA treatment up-regulated the expression of related genes in the AsA-GSH cycle, thereby maintaining higher AsA and GSH levels during cold storage and inhibiting the occurrence of chilling injury in peach fruit.

Many studies have found transcription factors such as ERFs have been identified to be involved in AsA metabolic pathway in Arabidopsis, tomato and citrus fruit (Zhang et al., 2009; Hu et al., 2016; Ye et al., 2019). *AtERF98* triggers the expression of genes involved in the D-Man/L-Gal synthesis pathway and the *MIOX4* gene in the myo-inositol pathway (Yuan et al., 2020). The function of *AtERF98*-regulated AsA synthesis was severely impaired in vtc1-1 mutants, suggesting that *AtERF98* is involved in the D-Man/L-Gal synthesis pathway and plays a vital role in regulating the synthesis of AsA (Zhang et al., 2012). In this study, we found that the expression of three *PpERF* genes was up-regulated in the peach GABA-treated group when compared with the untreated group, showing that ERFs might also participate in the metabolic regulation of AsA and GSH contents.

## Conclusion

In conclusion, after GABA treatment, the chilling injury of peach fruit was decreased. GABA increased AsA and GSH levels by regulating the expression of AsA and GSH-related genes in cold-stored peach. Therefore, we believed that GABA treatment could enhance chilling tolerance due to the increased levels of AsA and GSH. Furthermore, our results also suggested three candidate ERF transcription factors might be involved in AsA and GSH biosynthesis. However, more evidence is needed to determine the relationship of ERF transcription factors and their networks to hormone signalling and hormone-mediated stress responses, which may help provide insight into the transcriptional regulation of AsA-GSH metabolic processes in postharvest peach.

## Data availability statement

The original contributions presented in the study are included in the article/**Supplementary Material**. Further inquiries can be directed to the corresponding author.

## Author contributions

CZ, ZY and SC contributed to conception and design of the study. SJ organized the database. QL performed the statistical analysis. CZ wrote the first draft of the manuscript. LS, SL, WC and WD wrote sections of the manuscript. All authors contributed to manuscript revision, read, and approved the submitted version.
