# Supplementary material for: γ-Aminobutyric acid treatment induced chilling tolerance in postharvest peach fruit by upregulating ascorbic acid and glutathione contents at the molecular level
Source: Front Plant Sci. 2022 Dec 7;13:1059979. doi: 10.3389/fpls.2022.1059979 (PMC9768863; doi:10.3389/fpls.2022.1059979)
Supplement: Supplementary file 1 [file Table_1.docx]

**Table 1. Primer sequences used for RT-qPCR analysis**

| **Gene name** | **Forward Primer** | **Reverse Primer** |
| --- | --- | --- |
| *PpPMM* | CCATAGGAGGACAGATAA | GGTTCGTTCAGATTCATA |
| *PpGMP* | AATATGGAGTGGTGGTTAT | GGTTCAACAGGTATATTCC |
| *PpGME1* | GAATGTGTAGAAGGTGTC | CTATCTCAGCCATCTCAT |
| *PpGME2* | AAGCTGCCTATCCAGCACAT | GTCAGTGCCTTGTGCCTTCT |
| *PpGGP* | ATTGCTTCTGATGATAATGG | GCTTGTGTTAATGGTGTC |
| *PpGPP* | TGGTGCTGTGATTGTTAC | TTTGATGCTGCTACTCTTT |
| *PpGALDH* | TAAGAGCATTGATGAGAG | GAAGTCCTGTAATACCAA |
| *PpMXIO4* | GAGGAGTATAGCAAGTTG | CAATCTTCATTAGGATAGTCT |
| *PpGALUR* | AACAACTGCTCAGGTATC | GTTCTGCTTCATTCTTTCC |
| *PpAO2* | ATCTACTCCGTGACCAAT | CTCCTATGTTGAACTCCATT |
| *PpGCS1* | CTGACAACAATCGCACTG | AACATCAAGAGCATAATCAACAT |
| *PpGCS2* | TGGACTGATACTGACAAG | GAGCATATTCAACATACTTCT |
| *PpGS* | GATAGAGACGAACACAAT | GCAGGATTATTAGGAACT |
| *PpGR2* | GATGGTTCGTTGTCTCTG | CCTACTTCCTCCAATCCT |
| *PpDHAR2* | TTACAAGTTTCACCTCAT | ACAATCACATCAGAATCA |
| *PpMDHAR* | AGTGTTCCTGATGTATATGC | ATGCTCAACTCTTCTAATCTC |
| *PpGPX2* | TGACGGCATTAAGTGGAA | GCTAAGAGGAGTAGTTGTAGG |
| *PpAPX* | CCATCAAGCAACAGTTCC | AGTAATCTCAACAGCAACAAC |
| *PpERF4* | AACTTCCCTCTCCCTTCTGA | GCTCGGGCTCTGATTGTT |
| *PpERF106* | ATAATCTCGCCATCCATA | GACACTTGCTGAAACTTA |
| *PpERF115* | ATCCATCTCTCACTCTAT | TTGTCTCACTCCTCTATA |
| *PpTEF2* | GGTGTGACGATGAAGAGTGATG | TGAAGGAGAGGGAAGGTGAAAG |
